# Virtual Biopsy by Electrical Impedance Spectroscopy in Barrett’s Carcinoma

**DOI:** 10.1007/s12029-021-00703-0

**Published:** 2021-09-24

**Authors:** Sandra Blößer, Andrea May, Lukas Welsch, Michael Ast, Susanne Braun, Thomas Velten, Margit Biehl, Jonas Tschammer, Elke Roeb, Mate Knabe

**Affiliations:** 1 Department of Medicine II, Sana Klinikum Offenbach, Starkenburgring 66, 63069 Offenbach, Germany; 2 Department of Medicine I, Asklepios Paulinen Klinik Wiesbaden, Geisenheimer Strasse 10, 65197 Wiesbaden, Germany; 3 Stockert GmbH, Bötzinger Strasse 72, 79111 Freiburg, Germany; 4 Fraunhofer Institute for Biomedical Engineering (IBMT), Ensheimer Strasse 48, 66386 St. Ingbert, Germany; 5 Department of Gastroenterology, Justus Liebig University of Giessen, Klinikstrasse 33, 35392 Giessen, Germany; 6 Present Address: Department of Gastroenterology, Medizinische Klinik I, University Hospital, Goethe University, Frankfurt, Germany, Theodor-Stern-Kai 7, 60590 Frankfurt, Germany; 7 Institute for Medical Informatics, Justus Liebig University of Giessen, Rudolf-Buchheim-Str. 6, 35392 Giessen, Germany; 8 Institute of Pathology, Sana Klinikum Offenbach, Starkenburgring 66, 63069 Offenbach, Germany

**Keywords:** Esophageal carcinoma, Electrical impedance spectroscopy, Virtual biopsy, Barrett’s cancer

## Abstract

**Purpose:**

Early detection of adenocarcinomas in the esophagus is crucial for achieving curative endoscopic therapy. Targeted biopsies of suspicious lesions, as well as four-quadrant biopsies, represent the current diagnostic standard. However, this procedure is time-consuming, cost-intensive, and examiner-dependent. The aim of this study was to test whether impedance spectroscopy is capable of distinguishing between healthy, premalignant, and malignant lesions. An ex vivo measurement method was developed to examine esophageal lesions using impedance spectroscopy immediately after endoscopic resection.

**Methods:**

After endoscopic resection of suspicious lesions in the esophagus, impedance measurements were performed on resected cork-covered tissue using a measuring head that was developed, with eight gold electrodes, over 10 different measurement settings and with frequencies from 100 Hz to 1 MHz.

**Results:**

A total of 105 measurements were performed in 60 patients. A dataset of 400 per investigation and a total of more than 42,000 impedance measurements were therefore collected. Electrical impedance spectroscopy (EIS) was able to detect dysplastic esophageal mucosa with a sensitivity of 81% in Barrett’s esophagus.

**Conclusion:**

In summary, EIS was able to distinguish different tissue characteristics in the different esophageal tissues. EIS thus holds potential for further development of targeted biopsies during surveillance endoscopy.

Trial Registration

NCT04046601

## Background

In recent years, there has been a significant increase in the incidence of esophageal carcinomas in Western industrialized countries. Lifestyle trends in developing countries are expected to lead to a further rise in the incidence of the disease in the future [1, 2].

In contrast to advanced carcinomas, early carcinomas of the esophagus can be treated safely and with good results using local endoscopic interventions [1]. The outcome and prognosis depend on detection and treatment in the early stages of the disease. Preventive examinations of the esophagus and gastrointestinal tract should therefore be able to detect early or preliminary stages of the lesions.

Detection of intraepithelial neoplasia is the most valid marker for an increased risk of malignancy in existing Barrett’s esophagus. In most patients with Barrett’s esophagus who develop carcinoma, a linear progression from metaplasia to initially low-grade and then high-grade neoplasia can be observed.

### Existing State of the Art

Patients with known Barrett’s esophagus, warning signs, or a high level of familial risk undergo preventive examinations. During gastroscopy, undirected four-quadrant biopsies are taken in accordance with the Seattle protocol. This involves one biopsy being taken in succession at intervals of 2 cm in the esophagus at angles of 0°, 90°, 180°, and 270°, as well as from areas with a neoplastic appearance [3–5].

These methods, which are currently standard, have some disadvantages:

- They are time-consuming, expensive, and invasive for patients.
- The histological sample cannot be evaluated by endoscopists themselves, and an expert in histology is required.
- Histological evaluation cannot be carried out during the endoscopic examination. The patient needs to receive a new appointment for follow-up treatment if the findings are positive, and—in addition to time considerations for the patient and medical staff—this also entails further costs and risks (e.g., repeated sterilization of the endoscope, and possibly repeated anesthesia).
- After the first biopsy, the endoscopist’s vision is limited due to superficial bleeding. Thus, only untargeted samples can be taken.
- In patients with long-segment Barrett’s, the protocol is often not followed correctly, and this makes monitoring difficult [6].

Several new examination techniques for diagnosing neoplastic Barrett’s esophagus have been developed in recent years, including chromoendoscopy using various contrasting staining techniques, and also new optical imaging techniques for “virtual” chromoendoscopy, such as narrow-band imaging (NBI, Olympus), I-Scan (Pentax Medical), FICE (Fujinon Intelligent Chromoendoscopy, Fujinon), and confocal laser microscopy [7–9].

It would be desirable to have new, simple diagnostic procedures that are noninvasive but have validity comparable to that of a histological sample. Impedance measurement of esophageal carcinomas might be one option. By this new procedure, the local tissue is tested for electrical conductivity and compared with histological assessment by an expert pathologist.

Depending on its biological structure, biological tissue has a unique, complex electrical impedance [10, 11].

When electrical potential is applied to tissue, the current flows through the intracellular and extracellular spaces. Restricted mainly to extracellular spaces at low frequencies, the current is increasingly penetrating the membrane with higher frequencies and then reaching intracellular spaces as well [11].

Healthy esophageal mucosa is usually covered with layers of squamous epithelia. This type of epithelium is compact, with narrow spaces between, resulting in relatively high electrical resistance. In precancerous and inflammatory conditions, the integrity of the mucosa is altered and the extracellular spaces are expanded. This change can be assessed on the basis of a drop in resistance on impedance measurements [12]

The cell’s biological structure also changes continuously during the development of cancer, due to chromatin modification and increasing cell volume, leading to a reduction in the extracellular space. As the extracellular space declines, resistance to low frequencies that mainly flow through the extracellular space increases, resulting in a larger impedance in the low-frequency range [13]. These changes in electrical properties can be used to distinguish healthy tissue from neoplastic tissue. In addition to these criteria, the value of impedance measurement depends on the temperature, pressure, and type of material used for electrodes. In addition, the electrode size and distance also influence measurements, as well as suitable location and wiring of the electrodes.

## Methods

This joint project was conducted under the direction of the Department of Gastroenterology at Sana Klinikum in Offenbach, the Fraunhofer Institute for Biomedical Engineering (IBMT) in St. Ingbert, Stockert GmbH in Freiburg, and Justus Liebig University Gießen.

The objective of the project was to develop an in vivo measurement method and instrument for easy and cost-effective early detection of esophageal carcinomas. The aim is to use this probe to detect early tumors in the healthy esophagus. Proof of clinical efficacy was to be confirmed in a clinical study. The measurements were initially to be carried out ex vivo. The project originated in a publication by Knabe et al. demonstrating that there is a relationship between impedance measurements and the tissue being measured [14].

The test series were approved by the local ethics committee of the State Medical Association of Hesse, Germany (no.: FF 742,011), and all methods were performed in accordance with the relevant guidelines and regulations. The study is registered at clinical trials.gov as a prospectively study, registration number NCT04046601 and registration date on 06/08/2019.

Human tissue is removed during endoscopic resections that are planned in any case, and it is subsequently analyzed using impedance measurement. All patients received and signed an informed consent and a privacy statement prior to endoscopic resection.

**Fig. 1:**
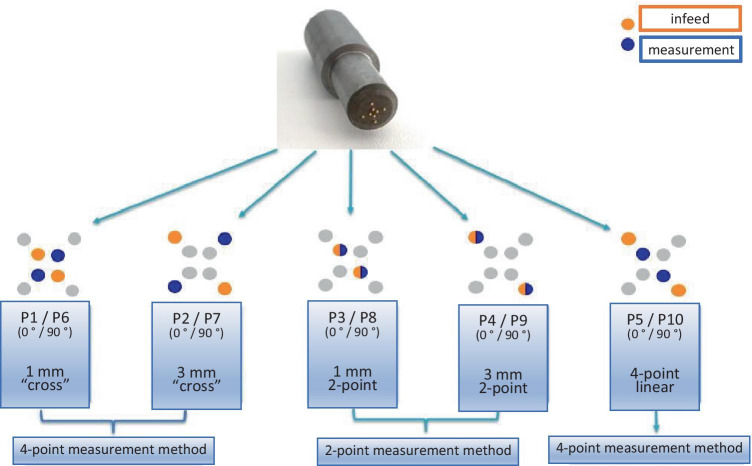
Measurement probe and interconnection principles

The aim of the test series was to establish whether there is a correlation between impedance measurements and pathological findings. In summary, the measurement procedure was as follows. The endoscopic resection was performed with a ligation device without subcutaneous injection. Immediately after removal of the target lesion, the resected samples were fixed on cork and had their electrical impedance properties examined using a pencil probe (diameter 5 mm) with eight gold electrodes with a diameter of 0.5 mm. Electrodes are arranged at the corners of two concentrical squares with a side length of 3 mm and 1 mm, respectively. The measurements were performed within a frequency range from 100 Hz to 1 MHz (10 measured values per decade) with an applied input potential of 20 mV, each with 10 different measurement principles (P1–P10), which are briefly explained in Fig. 1.

Two electrodes are used to feed in the impedance measurement signal. With the two-point measuring method, the same electrodes are also used to measure the voltage; with the four-point method, two other electrodes are used. The measuring principles P1–P10 differ in the electrode interconnection as well as in the measurement method (four-point linear measurement, two-point linear measurement, and cross measurement).

The impedance measurement depends on the tissue properties, contact pressure, temperature, and the distance between the electrodes. Different electrode arrangements thus result in a different depth measurement. The pressure is ensured by the weight of the measuring head. The area is then marked with tissue ink and photographed. All of the resected specimens were assessed by a local expert pathologist with long-standing experience in the field of esophageal cancer.

**Fig. 2:**
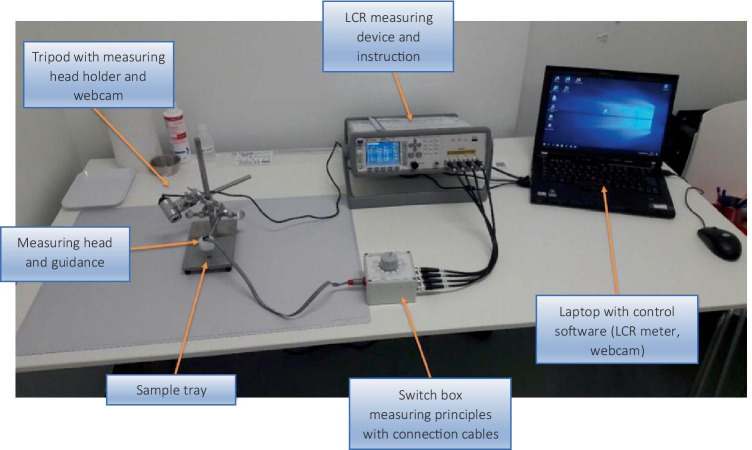
Setup of the measurement system

The experimental setup in the Sana Klinikum is shown in Fig. 2. To allow biologically unchanged resection specimens to be measured as quickly as possible, the setup was placed in close proximity to the intervention room.

## Outcomes

Table 1 summarizes the data that were collected. A total of 60 patients were included in the study (57 men and three women). The patients’ mean age was 66.77 years (minimum 48 years, maximum 91 years). Endoscopic treatment for Barrett’s esophagus was indicated in these patients due to previous or current neoplasia. Endoscopic resection was performed using the ligation technique without prior injection.

**Table 1:** Sample size and measurements

	*n*
Total patients	60
Total measurements	105
Evaluable measurements	105
Need for therapy	70
No need for therapy	35

Low-grade intestinal neoplasia (LGIN) and high-grade intestinal neoplasia (HGIN) were confirmed by a second pathologist. Table 2 shows the results of histological analysis of the measuring points examined.

**Table 2:** Pathological results after endoscopic resection

Histology	*n*	%
Barrett’s mucosa	15	14.3
Barrett’s, LGIN	20	19.1
Barrett’s, HGIN	22	20.9
Barrett’s, mucosal carcinoma	36	34.3
Barrett’s, submucosal carcinoma	12	11.4
Total	105	100.0

*HGIN*, high-grade intraepithelial neoplasia; *LGIN*, low-grade intraepithelial neoplasia

The group was subsequently divided into two subgroups, depending on the histological results:

- Barrett’s mucosa and LGIN
- HGIN and T1 carcinoma (m1–sm3)

Finally, the impedance values were compared with the histological results for each measurement, and the sensitivity, specificity, and accuracy were calculated. In a receiver operating characteristic (ROC) analysis, the cutoff value was carefully selected to obtain the highest possible sensitivity to the new procedure. Statistical calculations were carried out using IBM SPSS Statistics for Windows, version 23 (IBM Corporation, Armonk, NY) using ROC analysis and R [15] using the Wilcoxon test and Holm method for adjusting *p*-value.

The impedance of frequencies from 100 Hz to 1 MHz (divided into 40 frequency points) was determined using 10 different measurement principles (Fig. 1). A dataset of 400 per investigation and a total of more than 42,000 impedance measurements were therefore collected.

Using the Wilcoxon test and Holm method for adjusting the *p*-value, all frequencies and measuring principles were examined for significant differences in impedance between the group with or without treatment in Barrett’s mucosa.

## Results

A total of 105 examinations were carried out with 70 findings requiring treatment and 35 findings with no need for therapy. The following results were obtained.

**Fig. 3:**
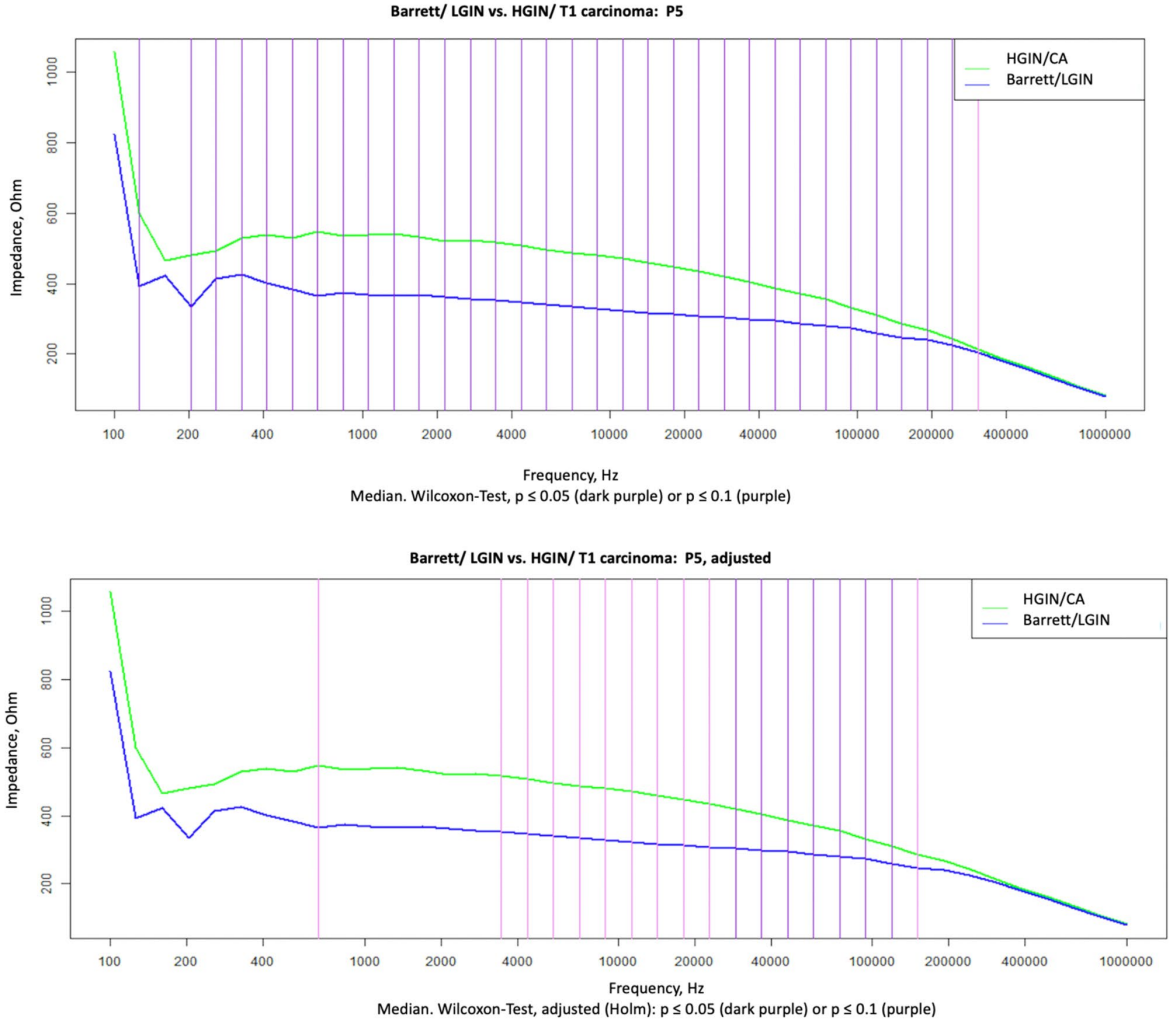
Wilcoxon test not adjusted and adjusted

For measurement method P5 (four-point linear, see Fig. 1), significant differences (*P* < 0.05) were observed for frequencies in the range of 203 Hz–307 kHz. After adjustment, *p*-values in the range of 28–151 kHz remained significant (Fig. 3). The method was thus able to distinguish between mucosa requiring treatment and mucosa not needing treatment.

With two further measurement settings (P3 and P8; two-point measurement method, see Fig. 1), there was also a significant difference (*P* < 0.05) in the Wilcoxon test over all frequencies, after adjusting with Holm only in P8 from 623 kHz to 1 MHz.

**Fig. 4:**
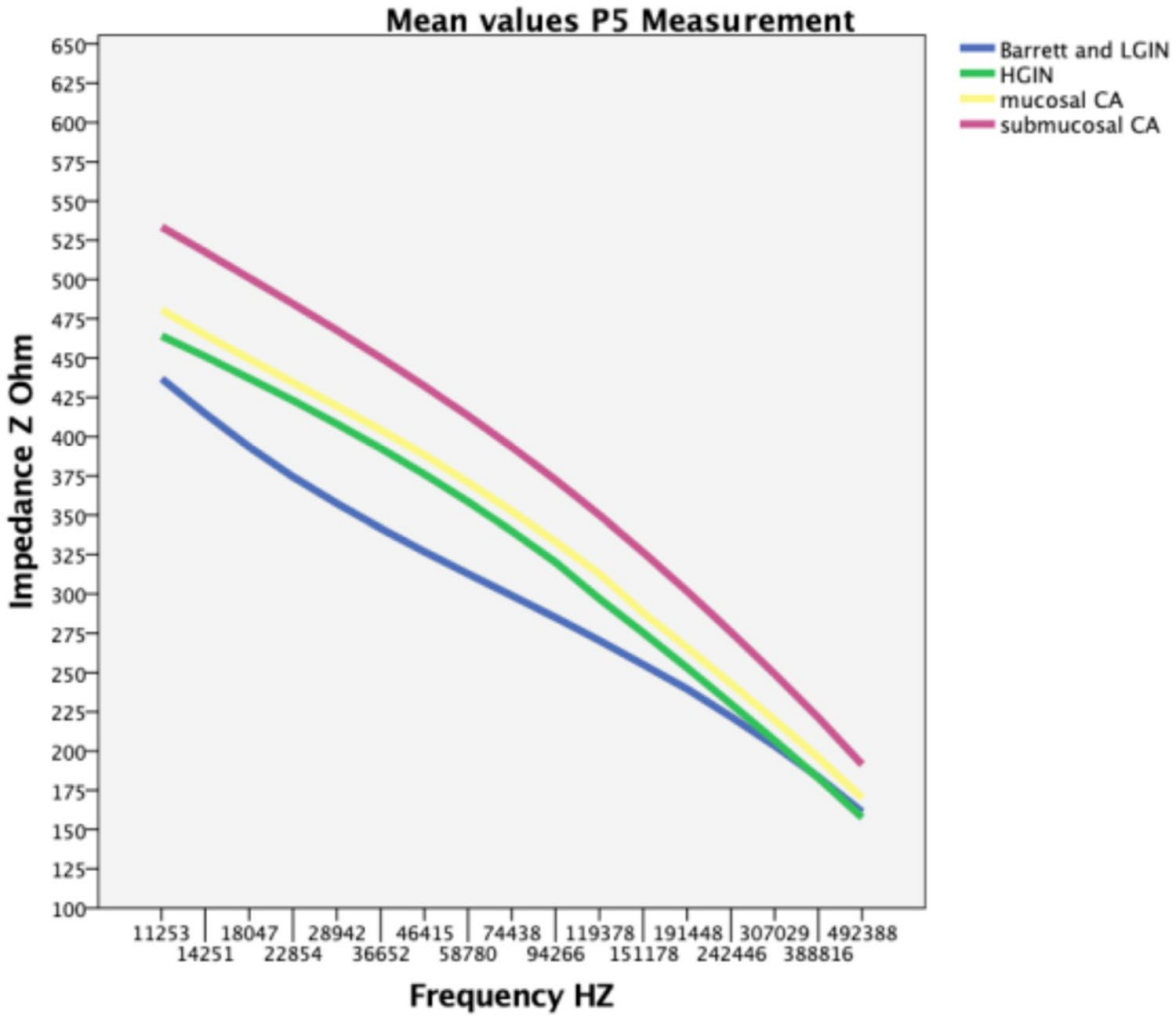
Mean values for P5 measurements

Table 3 lists the mean impedance values for different histological results over the most important frequencies in the P5 wiring. The significant areas are shaded in Table 3, and are also shown in Fig. 4, in which Barrett’s mucosa and LGIN appear in a range of significant measurements with significantly lower impedance than HGIN and carcinoma.

**Table 3:** Mean impedance values (Ohm) in the Barrett’s esophagus cohort. Significant areas are presented in italics

Frequency (Hz) P5	Barrett’s (Z)	LGIN (Z)	HGIN (Z)	Mucosal cancer (Z)	Submucosal cancer (Z)	Barrett’s and LGIN (Z)
22,854	323	415	423	434	485	374
*28,942*	*317*	*389*	*408*	*419*	*468*	*358*
*36,652*	*312*	*365*	*393*	*404*	*450*	*342*
*46,415*	*306*	*343*	*376*	*388*	*432*	*327*
*58,780*	*299*	*323*	*359*	*371*	*413*	*313*
*74,438*	*292*	*305*	*340*	*352*	*393*	*299*
*94,266*	*283*	*287*	*320*	*333*	*372*	*285*
*119,378*	*272*	*269*	*297*	*313*	*350*	*270*
*151,178*	*259*	*252*	*275*	*288*	*327*	*255*
191,448	245	236	253	266	302	240
242,446	228	218	230	243	276	222

*HGIN*, high-grade intraepithelial neoplasia; *LGIN*, low-grade intraepithelial neoplasia

**Fig. 5:**
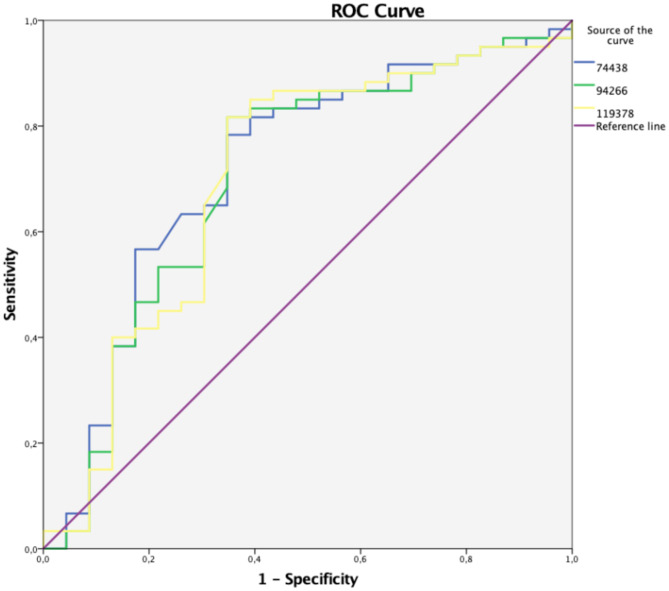
Receiving operating characteristics curve for P5 measurements

The ROC analysis of P5 measurements at 74 kHz, 94 kHz, and 119 kHz for HGIN/adenocarcinoma showed the best predictive potentials for the significant frequencies. A cutoff value with an impedance of > 316 Ω indicated a high probability of a diagnosis of adenocarcinoma. The sensitivity and specificity of the method were 81% and 61%, respectively (Fig. 5).

In a further evaluation, mucosal Barrett’s adenocarcinomas (m1–m4) and submucosal Barrett’s adenocarcinomas (sm1–sm3) were compared, without showing any difference in the Wilcoxon test.

## Discussion

In view of the prognostic and therapeutic relevance of early detection of malignant changes in the esophagus, various advanced diagnostic imaging techniques are being investigated. The absence of any clear recommendation for the routine use of such methods in monitoring patients with Barrett’s esophagus (BE) is partly due to the fact that their use in everyday practice has not yet been validated in large studies, or has not reached the threshold for monitoring patients with BE set by the American Society for Gastrointestinal Endoscopy’s Preservation and Incorporation of Valuable Endoscopic Innovations (PIVI) initiative [16]. Before the current Seattle protocol is replaced, the PIVI recommends that a targeted imaging technique with a sensitivity per patient of at least 90%, a negative predictive value of at least 98%, and a specificity of at least 80% should be used for detecting high-grade dysplasia or early adenocarcinoma.

As shown in the present study, impedance spectroscopy is capable of reliably distinguishing between nonneoplastic Barrett’s mucosa and neoplastic Barrett’s mucosa or adenocarcinoma in ex vivo tissue samples. This could provide investigators with a screening option or decision-making support for detecting neoplastic lesions in Barrett’s mucosa, which ideally might be able to reduce the number of biopsies that need to be taken.

On the basis of the best measuring points, a cutoff value with an impedance of > 316 Ω would most likely correspond to a diagnosis of adenocarcinoma, thus indicating endoscopic resection without biopsy. However, the PIVI requirements were not met in the present study (with sensitivity and specificity levels of 81% and 61%, respectively).

Comparative studies have shown that impedance spectroscopy is capable of detecting precancerous lesions in a wide range of different tissues. It has been found to distinguish reliably between benign and malignant tissue in the prostate, and is able to identify malignant melanoma, cervical cancer, and malignant tissue in the upper gastrointestinal tract [17–21].

All biological tissues have a frequency-dependent electrical impedance, as tissues contain both resistive and capacitive components (cells, matrix, etc.; charge storage). Both the size of the impedance and the dependency of the impedance on the frequency are related to the composition of the tissue, so that different tissue structures are associated with different frequency bands within an impedance spectrum. At high frequencies (> 1 GHz), the molecular structure is the determining factor, whereas at low frequencies (< 100 Hz), it is charge accumulation at large membrane interfaces that is predominant. At frequencies from several kilohertz to 1 MHz, known as the β-dispersion region, cell structures are the main determinant of tissue impedance. Within the β-dispersion region, low-frequency current flows around the cells, and resistance to the flow depends on cell spacing and the cellular arrangement of the tissue. At higher frequencies, however, electricity can penetrate the cell membranes and therefore both cells and extracellular spaces. Flow resistance is determined by intracellular volume and the size of the cell nucleus [22].

On the basis of these considerations, it should be possible to make clear distinctions between benign and malignant findings using impedance measurements. The present results thus show significant differences only in higher frequency ranges—from 28 kHz to 1 MHz—because a large proportion of the current flows through the cells rather than the extracellular spaces.

The greatest advance made so far in the application of impedance spectroscopy lie in the diagnosis of skin cancer. The Nevisense method was developed by SciBase in order to detect malignant melanomas. In a study by Malvehy et al., Nevisense had a sensitivity of 96.6% (256 of 265 melanomas) and a specificity of 34.4%. The positive and negative predictive values for Nevisense were 21.1% and 98.2%, respectively. The observed sensitivity for skin cancer without melanoma was 100% (55 of 48 basal cell carcinomas and seven squamous cell carcinomas) [23].

Bioimpedance measurement has also been used for diagnosis and screening of oral squamous cell carcinomas and precursor lesions. Murdoch et al. reported a significant difference in the electrical impedance spectroscopy findings between patients with cancer and high-risk neoplasia, in comparison with those with low-risk neoplasia and control individuals, but there were no significant differences between benign lesions and normal controls [22].

Impedance spectroscopy can also be carried out in cervical tissue to identify malignant and premalignant lesions [24]

A study of the upper gastrointestinal tract by Keshtkar et al. reported significant differences in vivo between malignant and benign gastric tissue. Impedances in the malignant tissue were lower than those in benign tissue. However, the ROC analysis, with a value of 0.57 AUC, did not show a good level of distinction between malignant and benign findings [25]. A possible source of error might have been the contact pressure used, which was not precisely defined or reproducible.

A good degree of distinction between malignant and benign findings was described in a study on the detection of bladder carcinomas using impedance measurement, also by Keshtkar et al., and in this report, the impedance in malignant findings was found to be higher than that in benign findings, as in the present results [26].

As a possible reason for differing results in relation to the impedance spectrum, it should be noted that some studies (like the present one) were performed on ex vivo preparations, while others used in vivo measurements. This certainly alters the cell composition and electrical conductivity. In addition, completely different epithelial types were investigated in the various studies (squamous epithelium versus columnar epithelium versus urothelium, etc.). Temperature differences between ex vivo and in vivo measurements can also lead to changes in impedance. A study on rabbits published in 2016 reported on impedance measurements in parathyroid gland and thyroid gland, showing significant differences in impedance between in situ and ex vivo measurements, with a relevant temperature difference (in situ 28.0–31.6 °C and ex vivo 12.0–18.4 °C). The ex vivo measurements showed significantly higher impedances than the in situ measurements. However, weak points in the study included the lack of data on probe contact pressure and the use of dead rabbits for the measurements [27].

In addition, as in the present study, prior endoscopic loop resection using a diathermy loop can certainly lead to cell damage, which alters the impedance spectrum in contrast to in vivo measurements. The time interval between endoscopic resection and impedance measurement also needs to be taken into account. Furthermore, the temperature of the tissue was not determined before the measurement, which can also lead to a change in the impedance spectrum as described above.

However, impedance spectroscopy has the potential to support physicians in the early detection of cancer. The comparatively small number of healthy measurements carried out in the present patient cohort and the lack of comparative measurements (healthy versus ill) in the same patient leave some questions unanswered. As these were therapeutic endoscopic resections and we did not remove healthy mucosa on purpose, the analysis only included 15 measurements in normal Barrett’s mucosa and 20 in LGIN, in comparison with 22 in HGIN and 48 in carcinoma. It would be helpful to have a comparison to normal mucosa of the same patient for each measurement of pathological tissue. However, from an ethical point of view, it was not possible to resect specifically healthy mucosa. To allow statistically reliable conclusions to be drawn, additional series of measurements with equally distributed histological cohorts and in vivo measurements will be needed in order to eliminate these interfering factors.

In summary, electrical impedance spectroscopy was able to distinguish between different tissue characteristics in the different esophageal tissues. It therefore holds potential for further development of targeted biopsies in surveillance endoscopy.

## Data Availability

Not applicable.
